# Supplementary material for: Aβ-Induced Alterations in Membrane Lipids Occur before Synaptic Loss Appears
Source: Int J Mol Sci. 2022 Feb 19;23(4):2300. doi: 10.3390/ijms23042300 (PMC8877175; doi:10.3390/ijms23042300)
Supplement: Supplementary file 1 [file ijms-23-02300-s001.zip › ijms-1580590-supplementary.pdf]

International Journal of *Molecular Sciences*

MDPI

Supplementary Figures

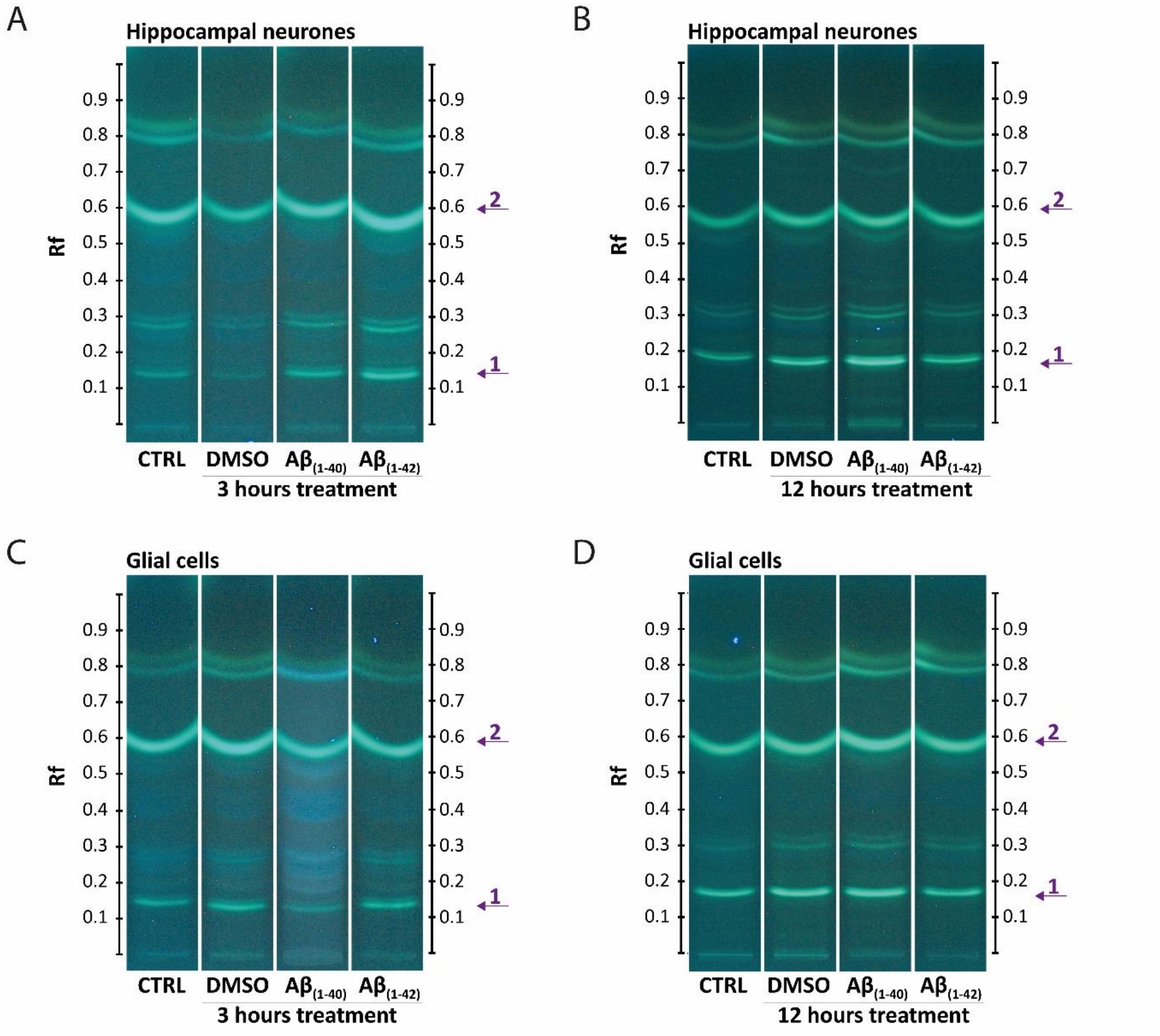

Figure S1. ***HPTLC-analysis based TopFluor lysophosphatidic acid (LPA) images of hippocampal neurones at DIV 12 and glial cells after 3 h and 12 h Aβ$_{(1-40)}$ and Aβ$_{(1-42)}$ treatment.*** LPA was used as an internal standard from control groups (CTRL and DMSO) and Aβ-treated groups (Aβ$_{(1-40)}$ and Aβ$_{(1-42)}$) at various time points of treatment (3 h and 12 h) of hippocampal neurones and glial cells ((**A**–**D**), all groups, from three independent experiments (N = 3)). 1μL/1 million cells fluorescent internal standard TopFluor LPA was added to each sample and fluorescence was detected with the CAMAG TLC Visualizer 2 at 366 nm. TopFluor LPA bound to the silica HPTLC plates at retention factor (Rf) around Rf 0.13 and Rf 0.59 (purple arrow 1 and 2).

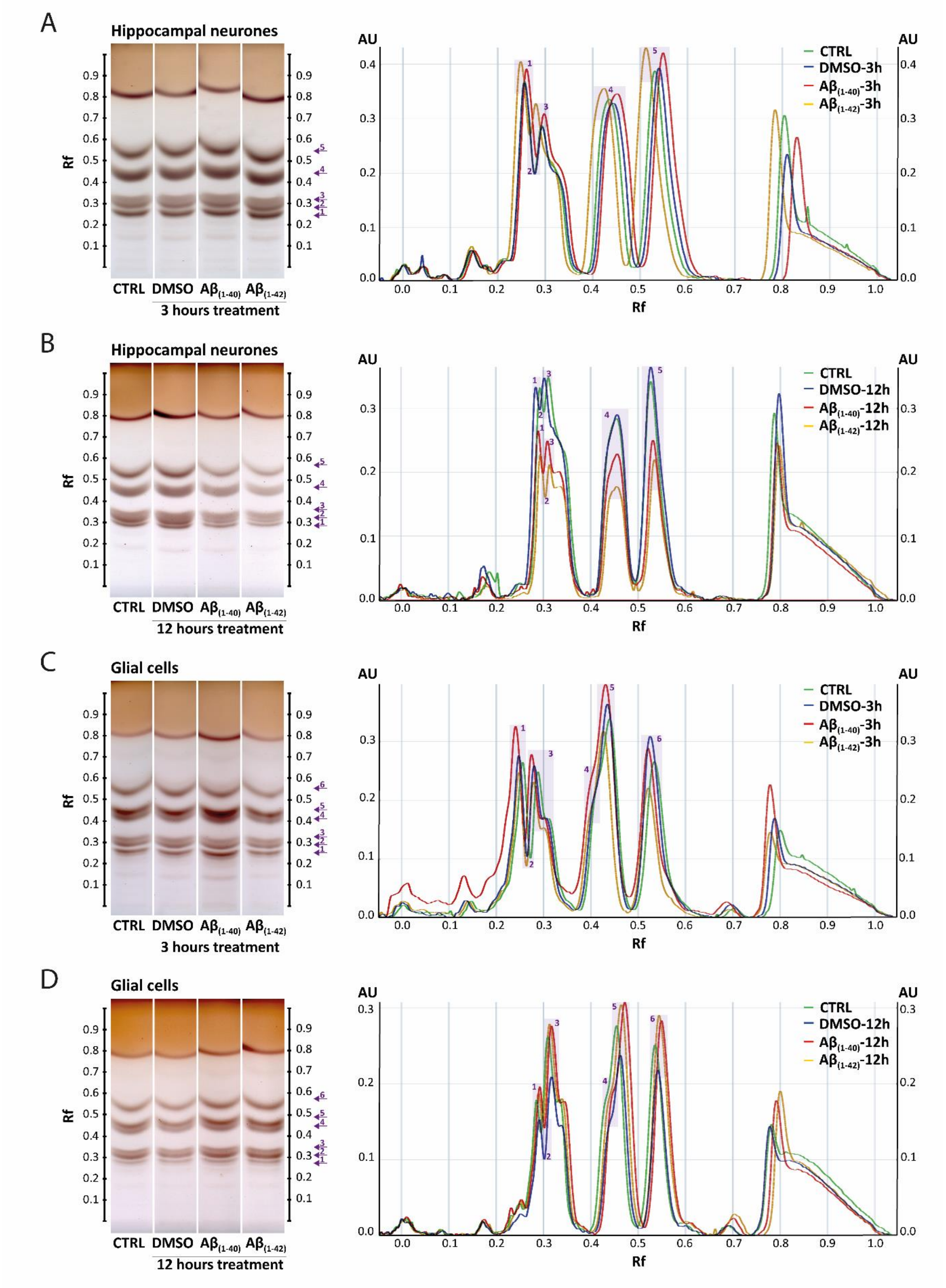

<u>Figure S2.</u> *HPTLC analysis based on derivatised copper (II)-sulfate images and scanning profiles of hippocampal neurones and glial cells after 3 h and 12 h of $A\beta_{(1-40)}$ or $A\beta_{(1-42)}$ treatment.* Derivatised copper (II)-sulfate images and scanning profiles that represent various lipid classes (sphingolipids and glycerophospholipids (1-6, purple arrow and boxes)) of control groups (CTRL and DMSO), Aβ-

treated groups ($A\beta_{(1–40)}$ and $A\beta_{(1–42)}$) at two different time points of treatment (3 h and 12 h) of hippocampal neurones at DIV 12 and glial cells ((**A**–**D**), all groups, from three independent experiments (N = 3). Changes in lipid classes on band intensity in derivatised copper-sulfate silica-gel HPTLC plate images are indicated by purple arrows (1-6), corresponding to their purple boxes (1-6) showing the digital copper-sulfate derivatised scanning profiles. Hippocampal neurones showed disregulated lipid classes of sphingolipids and glycerophospholipids (purple numbered arrows and boxes (1-5)) corresponding to the external standard (DOPC (4); DOPE (5); DOPS (1 and 2); 24:1 SM (3)) used. Glial cells showed disregulated lipid classes of sphingolipids and glycerophospholipids (purple numbered arrows and boxes (1-6)) in accordance to our external standard (DOPC (4 and 5); DOPE (6); DOPS (1 and 2); 24:1 SM (3)) used. Separated lipid classes represented by band intensity were calibrated in arbitrary units (AU) showing the relative absorbance of specific lipid classes. The retention factor (RF) represents the relative distance the sample ran compared to the distance the solvent front ran. TopFluor LPA was used as the internal loading control (Figure S1). (**A**) After 3 h, $A\beta_{(1–40)}$- and $A\beta_{(1–42)}$-treated hippocampal neurones showed slight increases both in band-intensity changes and lipid-profile graphs from different lipid classes compared to control groups. (**B**) After 12 h, $A\beta_{(1–40)}$- and $A\beta_{(1–42)}$-treated hippocampal neurones showed a prominent decrease in different lipid classes compared to control groups. Sphingolipids and glycerophospholipids showed a higher decrease after 12h $A\beta_{(1–42)}$ compared to $A\beta_{(1–40)}$-treated neurones. (**C**) Lipid classes in glial cells showed an increase after $A\beta_{(1–40)}$ treatment, while a decrease after $A\beta_{(1–42)}$ was visible at the early time point (3 h) compared to control groups. (**D**) An increase in lipid classes after 12 h was seen for both $A\beta_{(1–40)}$- and $A\beta_{(1–42)}$-treated glial cells compared to control groups.

Supplementary Tables

Table S1. List of retention factors (RF) and arbitrary unit (AU) of hippocampal neurones and glial cells (Figure S2).

Cell Types	Time Points	Numbered Arrows/Boxes	Conditions							
			CTRL		DMSO		$A\beta_{(1–40)}$		$A\beta_{(1–42)}$	
			RF	AU	RF	AU	RF	AU	RF	AU
Hippocampal neurones	3 h	1	0.257	0.367	0.257	0.364	0.260	0.390	0.249	0.403
		2	0.278	0.203	0.278	0.200	0.283	0.232	0.267	0.272
		3	0.293	0.287	0.294	0.284	0.298	0.308	0.280	0.325
		4	0.435	0.336	0.443	0.328	0.451	0.346	0.424	0.355
		5	0.531	0.388	0.539	0.393	0.549	0.420	0.513	0.429
	12 h	1	0.293	0.332	0.284	0.333	0.290	0.265	0.294	0.228
		2	0.301	0.294	0.292	0.297	0.300	0.198	0.305	0.160
		3	0.310	0.348	0.302	0.348	0.309	0.249	0.313	0.212
		4	0.455	0.284	0.456	0.290	0.456	0.229	0.455	0.178
		5	0.527	0.342	0.527	0.365	0.531	0.250	0.535	0.220
Glial cells	3 h	1	0.256	0.265	0.248	0.276	0.241	0.326	0.248	0.247
		2	0.272	0.101	0.265	0.104	0.260	0.131	0.264	0.088
		3	0.288	0.249	0.280	0.259	0.274	0.278	0.279	0.231
		4	0.408–0.424	0.206–0.265	0.407–0.420	0.206–0.265	0.394–0.409	0.206–0.265	0.406–0.416	0.206–0.265
		5	0.440	0.339	0.435	0.364	0.431	0.397	0.427	0.318
		6	0.534	0.266	0.526	0.309	0.521	0.288	0.521	0.221
	12 h	1	0.256	0.265	0.248	0.276	0.241	0.326	0.248	0.247
		2	0.272	0.101	0.265	0.104	0.260	0.131	0.264	0.088
		3	0.288	0.249	0.280	0.259	0.274	0.278	0.279	0.231

		**4**	0.408–0.424	0.206–0.265	0.407–0.420	0.206–0.265	0.394–0.409	0.206–0.265	0.406–0.416	0.206–0.265
		**5**	0.440	0.339	0.435	0.364	0.431	0.397	0.427	0.318
		**6**	0.534	0.266	0.526	0.309	0.521	0.288	0.521	0.221

Retention factors (RF) and arbitrary units (AU) for the different conditions (CTRL, DMSO, $A\beta_{(1-40)}$ and $A\beta_{(1-42)}$) after 3 h and 12 h treatment of 12 DIV hippocampal neurones and glial cells. Here, the RF and AU show the altered expression in different lipid classes (Figure S2, numbered arrows and boxes). Hippocampal neurons at DIV 12: purple numbered arrows and boxes refer to the following disregulated lipid classes (sphingolipids and glycerophospholipids) of our external standards; DOPC (4); DOPE (5); DOPS (1 and 2); 24:1 SM (3). Glial cells: purple numbered arrows and boxes refer to the following disregulated lipid classes of our external standards; DOPC (4 and 5); DOPE (6); DOPS (1 and 2); 24:1 SM (3).

Table S2. List of target ions/MRM transitions, assignment to internal standard and mode of analysis.

**Name**	**Internal Standard**	**Mode**	**Target ion Q1 > Q3**
C15:0 Cer		APCI	524.4 > 264.3
C12:0 Cer	>C15:0 Cer<	APCI	482.6>264.4
C14:0 Cer	>C15:0 Cer<	APCI	510.7>264.4
C16:0 Cer	>C15:0 Cer<	APCI	538.7>264.4
C18:0 Cer	>C15:0 Cer<	APCI	566.7>264.4
C18:1 Cer	>C15:0 Cer<	APCI	564.7>264.4
C20:0 Cer	>C15:0 Cer<	APCI	594.7>264.4
C22:0 Cer	>C15:0 Cer<	APCI	622.8>264.4
C24:0 Cer	>C15:0 Cer<	APCI	650.9>264.4
C24:1 Cer	>C15:0 Cer<	APCI	648.9>264.4
C12:0 DHCer	>C15:0 Cer<	APCI	484.6>266.4
C14:0 DHCer	>C15:0 Cer<	APCI	512.7>266.4
C16:0 DHCer	>C15:0 Cer<	APCI	540.7>266.4
C18:0 DHCer	>C15:0 Cer<	APCI	568.7>266.4
C18:1 DHCer	>C15:0 Cer<	APCI	566.7>266.4
C20:0 DHCer	>C15:0 Cer<	APCI	596.7>266.4
C22:0 DHCer	>C15:0 Cer<	APCI	624.8>266.4
C24:0 DHCer	>C15:0 Cer<	APCI	652.9>266.4
C24:1 DHCer	>C15:0 Cer<	APCI	650.9>266.4
C12:0 LacCer	>C15:0 Cer<	APCI	806.6>264.4
C16:0 LacCer	>C15:0 Cer<	APCI	862.7>264.4
C18:0 LacCer	>C15:0 Cer<	APCI	890.7>264.4
C22:0 LacCer	>C15:0 Cer<	APCI	946.8>264.4
C24:1 LacCer	>C15:0 Cer<	APCI	972.9>264.4
C12:0 HexCer	>C15:0 Cer<	APCI	644.6>264.4
C14:0 HexCer	>C15:0 Cer<	APCI	672.6>264.4
C16:0 HexCer	>C15:0 Cer<	APCI	700.7>264.4
C18:0 HexCer	>C15:0 Cer<	APCI	728.7>264.4
C20:0 HexCer	>C15:0 Cer<	APCI	756.7>264.4

C22:0 HexCer	>C15:0 Cer<	APCI	784.8>264.4
C24:0 HexCer	>C15:0 Cer<	APCI	812.9>264.4
d17:1 So1P		ESI	366.4>250.4
d18:1 So1P	>d17:1 So1P<	ESI	380.4>264.4
d18:0 Sa1P	>d17:1 So1P<	ESI	382.4>266.4
d17:1 So		ESI	286.4>268.4
d18:1 So	>d17:1 So<	ESI	300.4>282.4
d18:0 Sa	>d17:1 So<	ESI	302.4>284.4
SPC	>C17:0 SM<	ESI	465.4>184.1
C17:0 SM		ESI	717.5>184.1
C12:0 SM	>C17:0 SM<	ESI	647.7>184.1
C14:0 SM	>C17:0 SM<	ESI	675.7>184.1
C16:0 SM	>C17:0 SM<	ESI	703.8>184.1
C18:1 SM	>C17:0 SM<	ESI	729.8>184.1
C18:0 SM	>C17:0 SM<	ESI	731.8>184.1
C20:0 SM	>C17:0 SM<	ESI	759.9>184.1
C22:0 SM	>C17:0 SM<	ESI	797.9>184.1
C24:1 SM	>C17:0 SM<	ESI	813.9>184.1
C24:0 SM	>C17:0 SM<	ESI	815.9>184.1
C26:1 SM	>C17:0 SM<	ESI	841.9>184.1
C26:0 SM	>C17:0 SM<	ESI	843.9>184.1
C14:0 DHSM	>C17:0 SM<	ESI	677.7>184.1
C16:0 DHSM	>C17:0 SM<	ESI	705.8>184.1
C18:1 DHSM	>C17:0 SM<	ESI	731.8>184.1
C18:0 DHSM	>C17:0 SM<	ESI	733.8>184.1
C20:0 DHSM	>C17:0 SM<	ESI	761.9>184.1
C22:0 DHSM	>C17:0 SM<	ESI	799.9>184.1
C24:1 DHSM	>C17:0 SM<	ESI	815.9>184.1
C24:0 DHSM	>C17:0 SM<	ESI	817.9>184.1
C26:1 DHSM	>C17:0 SM<	ESI	843.9>184.1
C26:0 DHSM	>C17:0 SM<	ESI	845.9>184.1
28:0 PC	>34:0 PC<	ESI	678.5>184.1
30:0 PC	>34:0 PC<	ESI	706.6>184.1
30:1 PC	>34:0 PC<	ESI	704.5>184.1
32:0 PC	>34:0 PC<	ESI	734.6>184.1
32:1 PC	>34:0 PC<	ESI	732.6>184.1
34:0 PC		ESI	762.5>184.1
34:1 PC	>34:0 PC<	ESI	760.6>184.1
34:2 PC	>34:0 PC<	ESI	758.6>184.1
36:0 PC	>34:0 PC<	ESI	790.7>184.1
36:1 PC	>34:0 PC<	ESI	788.6>184.1
36:2 PC	>34:0 PC<	ESI	786.6>184.1

36:3 PC	>34:0 PC<	ESI	784.6>184.1
36:4 PC	>34:0 PC<	ESI	782.6>184.1
38:0 PC	>34:0 PC<	ESI	818.7>184.1
38:1 PC	>34:0 PC<	ESI	816.7>184.1
38:2 PC	>34:0 PC<	ESI	814.7>184.1
38:3 PC	>34:0 PC<	ESI	812.6>184.1
38:4 PC	>34:0 PC<	ESI	810.6>184.1
16:0 LPC	>17:0 LPC<	ESI	496.3>184.1
16:1 LPC	>17:0 LPC<	ESI	494.3>184.1
17:0 LPC		ESI	510.2>184.1
18:0 LPC	>17:0 LPC<	ESI	524.4>184.1
18:1 LPC	>17:0 LPC<	ESI	522.4>184.1
18:2 LPC	>17:0 LPC<	ESI	520.4>184.1
20:0 LPC	>17:0 LPC<	ESI	552.4>184.1
20:1 LPC	>17:0 LPC<	ESI	550.4>184.1
20:3 LPC	>17:0 LPC<	ESI	546.4>184.1
20:4 LPC	>17:0 LPC<	ESI	544.4>184.1
16:0 LPE	>17:1 LPE<	ESI	454.3>313.3
16:1 LPE	>17:1 LPE<	ESI	452.3>311.3
17:1 LPE		ESI	466.3>325.3
18:0 LPE	>17:1 LPE<	ESI	482.3>341.3
18:1 LPE	>17:1 LPE<	ESI	480.3>339.3
18:2 LPE	>17:1 LPE<	ESI	478.3>337.3
20:4 LPE	>17:1 LPE<	ESI	502.3>361.3
14:1 LPG	>17:1 LPG<	ESI	455.3>283.2
16:1 LPG	>17:1 LPG<	ESI	483.3>311.2
17:1 LPG		ESI	497.3>325.2
16:0 LPS	>17:1 LPS<	ESI	498.3>313.3
17:1 LPS		ESI	510.3>325.3
18:0 LPS	>17:1 LPS<	ESI	526.3>341.3
18:2 LPS	>17:1 LPS<	ESI	522.3>337.3
Lyso-PAF	>17:0 LPC<	ESI	482.3>104.2

List of lipid analytes with their corresponding target ions/MRM transitions for tandem mass spectrometry (MS). Target ions/MRM transitions are assigned to their internal standard and mode of tandem MS analysis (APCI and ESI). Abbreviations: atmospheric-pressure chemical ionisation (APCI); electrospray ionisation (ESI); ceramide (Cer); dihydroceramide (DHCer); lactosylceramide (LacCer); monohexosylceramide (HexCer); sphingosine (So); sphinganine (Sa); sphingosine 1-phosphate (So1P); sphinganine 1-phosphate (Sa1P); sphingomyelin (SM); dihydrosphingomyelin (DHSM); sphingosylphosphorylcholine (SPC); phosphatidylcholine (PC); lysophosphatidylcholine (LPC); lysophosphatidylethanolamine (LPE); lysophosphatidylserine (LPS); lysophosphatidylglycerol (LPG). For CER, SM, and (lyso-) phosphadityl compounds, the number of carbon atoms and the number of unsaturated bonds are given; for PC, the total number of carbon atoms as well as of unsaturated bonds are given.
